# New family medicine residency training programme: Residents’ perspectives from the University of Botswana

**DOI:** 10.4102/phcfm.v8i1.1098

**Published:** 2016-08-31

**Authors:** Deogratias O. Mbuka, Stephane Tshitenge, Vincent Setlhare, Billy Tsima, Ganiyu Adewale, Luise Parsons

**Affiliations:** 1 Department of Family Medicine and Public Health, University of Botswana, Botswana; 2 Private Family Medicine Centre, Maun, Botswana

## Abstract

**Background:**

Family Medicine (FM) training is new in Botswana. No previous evaluation of the experiences and opinions of residents of the University of Botswana (UB) Family Medicine training programme has been reported.

**Aims:**

This study explored and assessed residents’ experiences and satisfaction with the FM training programme at the UB and solicited potential strategies for improvement from the residents.

**Methods:**

A descriptive survey using a self-administered questionnaire based on a Likert-type scale and open-ended questions was used to collect data from FM residents at the UB.

**Results:**

Eight out the 14 eligible residents participated to this study. Generally, residents were not satisfied with the FM training programme. Staff shortage, inadequate supervision and poor programme organisation by the faculty were the main reasons for this. However, the residents were satisfied with weekly training schedules and the diversity of patients in the current training sites. Residents’ potential solutions included an increase in staff, the acquisition of equipment at teaching sites and emphasis on FM core topics teachings. They had different views regarding how certain future career paths will be.

**Conclusions:**

Despite the general dissatisfaction among residents because of challenges faced by the training programme, we have learnt that residents are capable of valuable inputs for improvement of their programme when engaged. There is need for the Department of Family Medicine to work with the Ministry of Health to set a clear career pathway for future graduates and to reflect on residents’ input for possible implementation.

## Introduction

Residency training programmes in Family Medicine (FM) have been in existence in other parts of the world for a number of years as reported in studies carried out in the United States^1^ and Saudi Arabia.^2^

The development of FM residency programmes in Africa is now a reality with the setting of new FM centres in central, East, and West Africa.^3^

The evaluation of those programmes in a SWOT (strength, weakness, opportunity and threats) analysis^4^ reported weaknesses and strengths that will be worth looking at and comparing with other new training programmes such as the University of Botswana (UB) Family Medicine residency. The FM at the UB is one of the newly created programmes in Southern Africa^3^ and is a 4-year programme with an additional provision for 2 years under the UB regulations for completion.

We have not been able to access literature regarding challenges faced by newly created Southern African training programmes despite the existence of a twinning programme amongst countries in Southern Africa.^3^ Therefore, the reasons for this lack of published data on experiences of new FM training programmes in Southern Africa are not known.

The establishment of the first medical school in Botswana was motivated by a number of reasons.^5^ These included the fact that shortage of medical doctors (MD) within the Botswana health system was high and inequitable distribution of doctors within the country existed with rural areas experiencing more shortage compared to urban areas.^5,6,7^ FM was seen to have an important role in strengthening the health system,^8^ and the recommendation of the World Health Organisation on primary care reform^9^ attested to the need for FM training in Botswana. In addition, the overall effectiveness of family physicians (FPs) in some African countries^10,11^ highlighted the need for such training in Botswana.

To address the shortage of MDs in general and FPs in particular with the hope of addressing the health needs of the population, the government of Botswana through the UB started training for Master of Medicine (Internal Medicine and Paediatrics with Adolescent Health) in 2010, while other programmes for Master of Medicine (Family Medicine, Anaesthesia and Emergency Medicine) started in 2011.^5^ The first graduates of Masters programmes were expected in 2014, while those for FM and other programmes started in 2011 were expected in 2015.

Formative evaluation reported in studies done elsewhere using residents’ opinions to adjust the FM training programme have proven to be helpful.^12,13^ Therefore, it was important to identify the strengths and weakness of the UB FM residency programme similarly in order to help the UB Department of Family Medicine and other stakeholders to address issues and improve the training programme.

### Context of training

**FIGURE 1:**
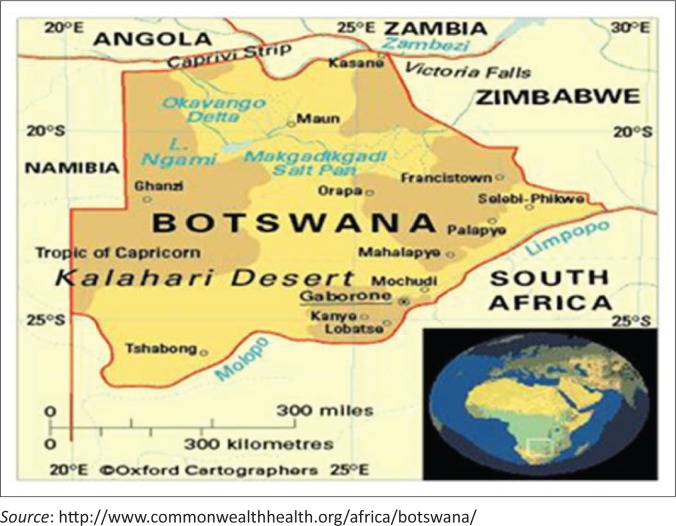
Botswana map with the three Family Medicine training sites namely Gaborone (and surroundings), Mahalapye and Maun.

When this research was done, the Department of Family Medicine at UB had three teaching sites, namely, Mahalapye (about 198 km from Gaborone), Maun (about 900 km from Gaborone) and Gaborone (Figure 1).

The head of the Department of Family Medicine operates from the main campus in Gaborone; consequently, communication between the three teaching sites has been challenging because of the distance between the sites. These three sites communicate through Skype©, telephone and email, while the preferred face-to-face meetings are infrequent.

At its inception in 2011, the FM residency programme had an initial resident–faculty ratio of 4:1, which deteriorated to 6:1 and 8:1 in Mahalapye and Maun, respectively, over time. The number of residents in the sites under study (Maun, Mahalapye) was four per each site in 2011, eight and six per site in 2012, respectively, in Maun and Mahalapye. These numbers remained unchanged till 2015 as there was no new intake of residents in 2013 because of acute staff shortage. The resident intakes from 2014 to 2015 were based in hospitals around Gaborone where the head of department was based. The number of lecturers in these sites was not stable as three of the appointed lecturers either resigned or did not apply for renewal of contract for personal reasons over time, while another member was enrolled to a 2-years master’s programme abroad. This resulted in a drop from the best number of seven lecturers to the lowest number of three (one per site in Maun and Mahalapye where the study was conducted, and the head of department in Gaborone), with an unchanged number of residents in Mahalapye and Maun. Thus, there was an increase in faculty workload between end of 2013 to early 2015, with reduction of staff and arrival of continuing two streams of undergraduate students in Mahalapye and Maun. The undergraduates were Bachelor in Medicine, Bachelor in Surgery students in their third and fifth year, doing 8-week rural exposure rotations in groups of 6–10 students, and they required supervision and teaching by faculty in the Department of Family Medicine. Possible solutions for this acute staff shortage from the Department of Family Medicine’s point of view were possible closure of existing separate training centres and relocation to Gaborone in order to optimise use of the few facility. However, this did not materialise. The faculty–resident ratio subsequently improved to the current 1:4, with the appointment of three new staff members from June 2015, and it is hoped that the growing residents’ complaints regarding inadequate supervision will be partly addressed.

The Department of Family Medicine uses a curriculum that was adapted from the Stellenbosch University (SU) training programme. In the context of Botswana, this consisted of the first 24 months training being based in district hospitals, and this consists of rotations in different specialties called ‘clinical domains rotations’ such as internal medicine, surgery, obstetrics and gynaecology, anaesthesia and paediatrics as indicated in Table 1. The subsequent 24 months of primary care/clinic-based placement are not represented in the table.

**TABLE 1:** Clinical domain rotations in Family Medicine and durations for the first 24 months.

Clinical domain rotations	Durations
General internal medicine	12 weeks
Obstetrics and gynaecology	12 weeks
Paediatrics	12 weeks
Accident and emergency	12 weeks
Anaesthetics	12 weeks
General surgery	12 weeks
Orthopaedics	12 weeks
Psychiatry (mental health)	4 weeks
Infectious disease control centre	4 weeks
Ear, nose and throat	2 weeks
Ophthalmology	2 weeks

*Source*: Authors’ own work

At the time of this study, the supervision during the hospital-based rotations was mainly done by the specialists employed by the Ministry of Health (MOH), and the primary care/clinic-based placement was supervised by faculty members who were FPs.

UB provides mandatory common core modules to all the residents regardless of discipline of specialisation within the Faculty of Medicine (FOM). These are communication skills, ethics and professionalism, public health and international health, research methods and introduction to the medical literature review. The FM academic activities include a monthly journal club and weekly tutorials consisting of case presentations, video viewing of consultations, clinical skills demonstrations, presentations of common problems encountered in primary healthcare and consultation skills discussions, which take place during tutorials and at the bedside. In the context of FM training in Botswana, continuing medical education presentations by residents, other health professionals and faculty members are also part of the teaching platform, conducted in the hospital meeting room for the benefit of the entire district hospital staff.

To assure quality training in the new Department of Family Medicine, benchmarking was done with SU, and Limpopo (Now Sefako Makgatho University of Health Sciences), and accreditation with the College of Family Physicians of South Africa was sought and granted.

### Aims of the study

This study assessed the level of satisfaction of residents with the FM training programme at UB, Botswana, and solicited from them possible solutions for improvement of the FM training programme.

### Contribution to field

Findings of this study may be the first to be reported among new Southern African FM training programmes about the strengths, weaknesses and potential solutions that any new training programme is likely to face. This study may be considered as a contribution to the conversation about challenges and potential solutions of starting FM programmes and in newly created FM training sites in southern Africa. It is hoped that, this study will encourage a collaborative regional effort to overcome common problems in the teaching of FP through information sharing and similar multicentre studies for regional solutions to challenges in FM training programmes.

## Methods

### Study design

This was a descriptive survey using a structured self-administered questionnaire.

### Setting

The study was done at Maun and Mahalapye FM training sites where two district hospitals of about 260 beds each, including surroundings clinics are used for practical and theoretical training of residents in FM.

### Study population and sampling strategy

All 14 eligible FM residents based in Maun and Mahalapye in December 2014 were invited to participate in the study. Six residents were based in Mahalapye and eight were based in Maun. During the study, the residents were either in their fourth or fifth year of training. The Department of Family Medicine did not have residents in the third year when the study was done, as during 2013 we did not recruit because of an acute shortage of staff.

### Data collection

A presentation on the research project (information session) to all residents at both sites (Mahalapye and Maun) was done in December 2014. This was followed by a collective email, with the consent form and questionnaire attached, sent out to the residents. The self-administered graduate medical education (GME) survey questionnaire,^13^ adapted to the Botswana setting, was used. The choice of the GME questionnaire was based on the availability of questions ready for use, and the ability of these questions to assess the context, inputs, process and output (CIPP). A more detailed questionnaire generated from a CIPP fomat^14^ accessed after the design will be considered for use in a future evaluation of the FM programme. To complement the GME questionnaire, new questions using the same style were added to fit the Maun and Mahalapye settings. The questions were not considered to be major distortions to the validated questionnaire as none of the original questions was altered. Researchers who were lecturers of the participants in the two sites collected the filled questionnaires from labelled boxes at designated places in the two sites where it was agreed that forms were to be privately dropped.

Data were collected through answers on a Likert-type scale, which assessed the level of satisfaction of residents regarding the content of the FM programme, the quality of teaching rendered by supervisors, the quality of feedback and assessment and their satisfaction with their work environment and teaching sites. The open-ended questions component captured participants’ opinions on strengths and weaknesses of the FM programme, residents’ opinions on the future career as FP in Botswana and recommendations for improvement.

As a result of slow uptake, the initial two months planned for data collection were extended. A monthly reminder was sent out by email and verbally during the three months resulting in approximately a five -month data collection period, to ensure maximum participation of residents. Unfortunately, participation remained low regardless of efforts to get everyone to participate.

### Data analysis

Quantitative data were captured in IBM SPSS 21 for statistical analysis. Frequencies were used to summarise the categorical variables. Answers to open-ended questions were categorised based on types of comments or solutions from the eight participants on a particular question. The two on-site researchers separately looked at data for possible themes and categorisation of comments. Final themes and categories were obtained after reconciling their findings, which were referred to as emerging themes. The process consisted in identifying and grouping similar ideas and answers into positive or negative categories with regard to strengths, weaknesses and solutions. Researcher triangulation was done by comparing findings from the two researchers after the review of same data set, and consensus was reached by discussion where divergent findings were noted. Of the eight respondents, the open-ended section of participant number seven was empty and was not included in the analysis. All eight questionnaires were completed in terms of the Likert-type scale section.

The justification of the choice of training sites, the strengths and weakness of the programme, residents’ opinions on their future career path and recommendation for programme improvement were analysed and reported.

### Ethical considerations

This study was done after being granted permission by the UB institutional review board (UB IRB; reference: URB/IRB/1469) and the MOH (reference: PPME 13/18/1 VIII (538)).

## Results

Of 14 eligible residents, 8 residents in their fourth or fifth years in the FM programme participated in this study. Three participants out of six were from Mahalapye and five out of eight were from Maun, making the total participation of more than half of eligible FM residents.

### Profiles of participants

Five participants were from the Maun training site while three were from the Mahalapye site. There were five male (5/8) and three female (3/8) participants, aged between 31 and 37 years, of whom four were married (4/8) and four were single (4/8). Half of the participants (4/8) were in the fourth year of training.

### Residents’ satisfaction with FM training programme

All participants agreed that they were satisfied with the diversity of patients and diagnoses in the training sites (8/8).

**FIGURE 2:**
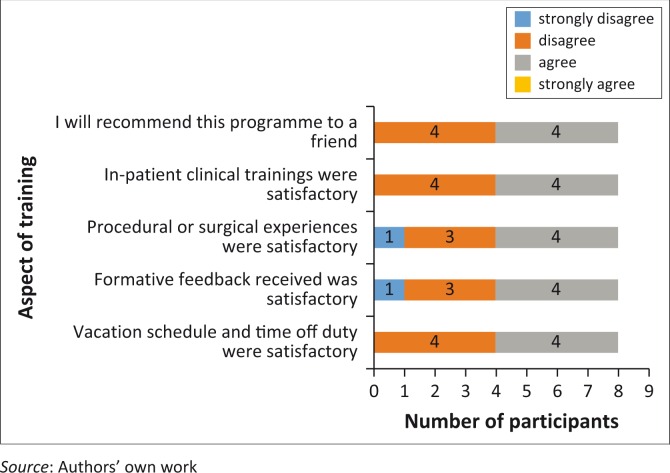
Number of participants in agreement or disagreement with satisfactory experiences from different aspects of the training in family medicine.

Over some aspects of the training programme, participants had divided views (4/8), with half of them reporting dissatisfaction while the other half were satisfied with the programme. These aspects are summarised in Figure 2 with the number of participants agreeing or disagreeing to have satisfaction with aspects of training programme, shown in the bar chart. In-patient clinical teaching setting, the procedural surgical experiences, the formative feedback received for performance improvement, the scheduling of vacation and time off-duty and whether residents were to recommend the programme to friends are reported in the Figure.

Most participants were not satisfied with other aspects of the training programme. They expressed particular dissatisfaction (number of residents dissatisfied/total of residents) with the following aspects of the programme:

- the programme organisation (7/8);
- the overall dissatisfaction with the programme (5/8);
- the teaching sessions (6/8);
- the guidance and mentorship (5/8);
- the performance evaluation (5/8); and
- the duty roster/work schedule (4/8).

**FIGURE 3:**
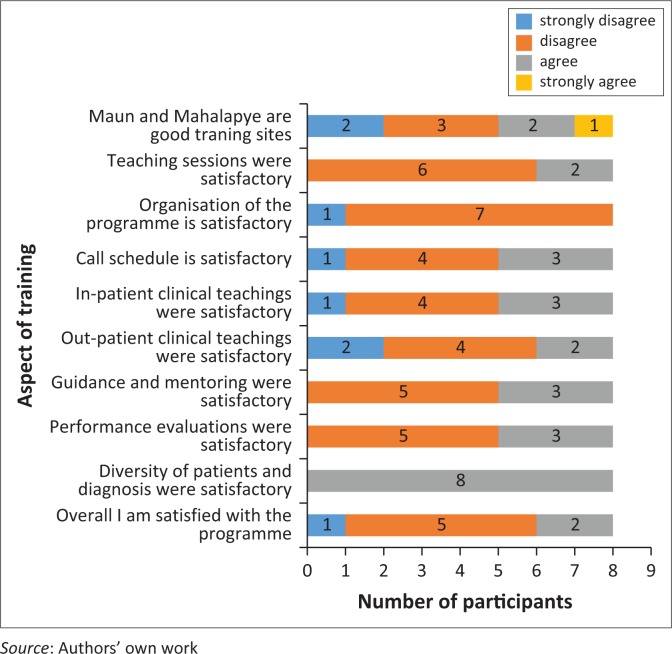
Number of participants in agreement or disagreement with satisfactory experiences from different aspects of the family medicine training programme.

Figure 3 is a colour-coded bar chart summarising the areas of agreement and disagreement regarding the satisfaction with the FM training in Botswana from the participants’ perspectives.

### Strengths of the programme

Residents viewed strengths of the programme as the support from different stakeholders, staff commitment, regular tutorials and protected study time.

#### Stakeholders’ support

Most participants considered the strength of the programme to be the support from regional universities, the support from private funders and the availability of infrastructure provided by the MOH:

‘Support from Stellenbosch and a sponsor like MEPI (Medical Education Partnership Initiative) as well as the ministry of health …, numerous available clinics can host us.’ (P1)

‘[*The*] collaboration with RSA universities …, [*and*] availability of internet services.’ (P3)

The valued smooth running of the online SU examinations, from the first year to the third year of the residency programme:

‘exams from first to third year seem to have been run well up to now.’ (P2)

#### Commitment of faculty staff

Some participants considered the commitment of the skeleton staff on the ground to be part of the strength of the programme:

‘Supportive and committed staff.’ (P6)

‘Committed staff.’ (P8)

#### Regular tutorials and time to study

Protected time and regular tutorial were considered by some as strengths of the programme as it allowed interaction with faculty and colleagues:

‘Protected study time and Wednesday’s classes [*tutorials*].’(P4)

### Weaknesses of the programme

The shortage of staff, inadequate supervision, too little focus on FM topics, poor departmental administration and a lack of faculty experience were reported by the majority as weaknesses of the programme. Other weaknesses that were mentioned were non-competitive faculty remuneration that caused difficulty in attracting and retaining staff, current training sites not being ideal, too heavy workload for residents, the lack of differentiation of roles for different levels of residents and insufficient equipment for training.

#### Staff shortage leading to inadequate supervision

Most participants viewed the shortage of staff and the inadequate supervision to be weaknesses of the programme:

‘Lack of human resources-lecturers.’(P3)

‘Shortage of staff is the main problem.’(P5)

The shortage of staff was differently expressed in terms of the clinical involvement of faculty and inadequate supervision:

‘Because as it stands they are not actively involved in clinical medicine with residents unless if they come to assess, obviously that ties with a point I made above that there is serious shortage of staff that need to be increased.’(P2)

## Lack of supervision

A participant expressed that the lack of supervision was the weakness of the programme while another considered the inability of recruiting more staff because of non-competitive salaries as a possible explanation for shortage of staff and inadequate supervision:

‘The lack or inability to recruit staff due to money reasons.’(P3)

‘Lack of supervision at inpatient and outpatient areas.’(P4)

### Departmental administration and faculty experience

Not having a FM lecturer with previous teaching or administrative experience in a medical school was seen as another weakness of the programme:

‘I also feel it is a blunder that amongst all our lecturers there is no one with prior teaching experience or having been part of administrative staff in running of a medical school that is why a lot of things are not going right.’(P2)

The same participant referred to the failure of producing necessary documents for residents in time as an administrative weakness of the programme while another referred particularly to the inexperience and possible lack of awareness of UB regulation as the cause to the weakness of the programme:

‘Poor administration of the faculty and inability to produce results in time.’(P2)

‘Staff inexperience and unawareness of UB standard procedures and poor support for staff from SOM [school of medicine] management.’(P8)

### Ideal training sites

Participants were divided as to whether Maun and Mahalapye were good training sites, with less than half of the participants favouring the sites. A participant thought that the current district hospital location of the training is part of the weakness of the programme, preferring a primary hospital location for training while a colleague was of the opposite opinion, favourable to district hospital:

‘Training in a district site which I believe is not appropriate for family medicine training; personally I believe it will be better in a primary hospital.’ (P2)

‘Good sites locations [are] district hospital.’ (P8)

### Work load, differentiation of tasks and equipment for training

Limited medical equipment at training sites and overworking were considered to be weaknesses of the programme as they did not promote procedural clinical skills which should be residents’ second nature.

‘Patient overload, limited equipment and tools …, we need to acquire skills so that they become second nature …, we also need a skills lab with manikins to practice on.’ (P1)

Another resident pointed out that the programme failed to consider progression in seniority with number of years in the programme and there was no point at which a resident was considered senior to medical officers. Faculty were perceived to be not very concerned about defending residents’ primary goal of learning as opposed to working in the hospital:

‘We work as medical officers from first year to the last year and faculty has totally allowed the hospital to do as it wishes with residents, providing service at total compromise of our learning forgetting that our true mandate is learning.’ (P2)

### Teaching and assessment

Participants perceived the lack of focus in teaching FM concepts or topics and the preparation towards the final year examination as inadequate and weakening the programme:

‘Lack of proper training in family medicine concepts.’ (P4)

‘No proper preparation for the exit exams.’ (P4)

### Potential solutions

Following were suggested:

#### Increase of faculty number and monetary incentives

Different participants viewed monetary incentives and staff increase as part of the solution to the current situation:

‘Increase of staff, remuneration for part-time lecturers.’ (P6)

‘A joint lecturer appointment to improve lecturer-resident supervision and monitoring.’ (P8)

‘Increase supervision and staff so that the residents get exposed to the everyday environment of a family physician.’ (P1)

#### Yearly plan and mock examination preparation

This comprises a yearly plan of activities, including mock preparation contrary to a 6-month plan that is being produced currently:

‘Please plan the year properly, and have mini mock exams at least x2 per year [about], different procedural skills we are expected to know.’ (P4)

To help administer FM programme, it was suggested the alignment of the programme with other UB master’s degree programme:

‘Align program with other UB masters courses and the use of same resources.’ (P8)

#### Collaborative teaching effort within the University of Botswana

From the collaborative teaching experience with public health, a participant thought that intensifying teaching collaborations with other UB departments will be beneficial for residents:

‘Make use of other UB department in teaching us things like public health and research.’ (P2)

#### Acquisition of more equipment for skills training

Some participants believed that acquiring on-site medical and simulation equipment will improve their skills which will help to provide quality care:

‘Have a skills lab, make sure we have basic instruments to enable us to manage patients according to evidence based standards.’ (P1)

‘Avail medical equipment that may be useful in helping residents acquire certain skills.’ (P2)

#### Teaching more family medicine topics

A participant suggested a possible solution that will help to have available adequate FM topics in the department:

‘Draw a syllabus for a longer module on family medicine concepts.’ (P8)

#### Flexibility of the programme

A participant thought that based on personal experience in the medical field residents should be allowed to spend more time in areas of weakness than in areas of strength, and therefore suggested:

‘Letting residents spend more time in the disciplines they have more inadequacies in, rather than forcing everyone to do same length of time in each in each discipline.’ (P2)

### Residents’ opinions on the future career path as family physicians in Botswana

Residents had mixed feeling about their future varying from uncertainty on career path to a bright future full of hope while two participants did not express their opinions on this.

The uncertainty was supported by:

‘I still currently have no confidence in Family Medicine concepts, and I still don’t know where we will work when we finish.’(P4)

‘A poor family physician deprived of opportunity to learn because of poor structure of programme …’ (P2)

‘Immediately after graduating will leave University and look for green pasture unless salary improves.’(P5)

The optimistic views were supported by:

‘I feel graduates are likely to improve the medical practice in Botswana by acting not only as doctors but also as mentors and managers to other health professionals … there is a bright future for graduate.’(P1)

‘Vast opportunities.’(P8)

‘Being able to function as medical manager.’ (P3)

## Discussion

In general, participants expressed dissatisfaction with the FM training programme. The shortage of staff was perceived to be a cause of many other unsatisfactory areas of FM training, while the only area of the programme with which all participants were satisfied was the exposure to a diversity of patients and diseases at both training sites.

The dissatisfaction with the supervision found in this study may have been because of inadequate staffing as the faculty–residents ratio dropped to 1:8 and 1:6, respectively, in the Maun and Mahalapye sites for more than a year. This situation has fortunately been corrected during the second half of 2015. Adequate staffing may address many weaknesses of the Botswana FM programme such as inadequate supervision of residents and inadequate staff to facilitate examination preparation.

The role of adequate supervision in FM residency is vital in learning facilitation as reported in a study in Saudi Arabia,^14^ and this gives credence to participants’ thoughts on this issue. However, inadequate supervision has been reported in different settings outside Africa,^12,15^ including in other new FM programmes in Africa implied by the lack of FPs and local teachers.^4^ In the case of UB, a FM programme which adopted a decentralised approach with inadequate staff in different training sites and difficulty in recruiting and retaining experienced staff necessitated consideration of growing our own staff, both the initial inexperienced staff and the graduates of the programme. Not doing so might endanger the development of FM in Botswana.

However, the closure of the two rural sties contemplated by the UB FM programme in response to the acute staff shortage experienced was not supported during the first national FM conference,^16^ which observed that a development of centres around Gaborone should be done keeping in mind staffing situation, while maintaining the existing two rural sites to ensure the spread of FM in Botswana. We have now four training centres in total, two of which are in villages near Gaborone.

The administrative inexperience of the staff members was another aspect of dissatisfaction. At the time of this research, it was still difficult to recruit experienced staff. This is a common experience among low-income countries in the region but a high middle-income country like Botswana experienced similar challenges; this is why the solution may be for these new FM departments in the African region to grow from within and to develop regional collaboration for staff development.

Poor organisation of new FM training programmes has been reported elsewhere^12,15,17^ and this needs to be addressed in the UB programme. A suggestion that the Botswana FM programme should be aligned to other master’s programmes at the UB is perhaps a good place to start. In fact, at the time this study took place, the FM programme and other FOM master’s degree programmes were the only ones to start their year in January while other older masters’ degree programmes in UB start in August, and this has implications on administrative issues.

The concern that there should be more focus on FM-oriented topics was similar to the experiences of Turkish trainees^18^ who missed FM topics during their hospital rotations. However, Japanese trainees^17^ actually felt deprived of adequate clinical teaching during their FM programme. This shows the need of a balanced emphasis in the curriculum of the Botswana FM programme, namely between the clinical procedural skills in general and the FM concepts and special consultation skills that mould a good FP.

Participants found the workload excessive and incompatible with their training. This experience is similar to findings in FM training programmes in some African countries.^4^ This situation may be because of the inherent workload in primary care settings in Africa and in residency programmes in general. Balancing workload and protected learning opportunities in the FM residency programme is tricky because significant workload is itself a good preparation for future work as FP and a leader in the primary healthcare setting.

The limitations of this study included the small sample size, the low response rate. This means that residents ‘perceptions of the University of Botswana FM Master’ programme requires further exploration. However, indications are that some of these findings could be transferable to other similar settings,^19^ as shown by similarities with findings from other African countries following a SWOT analysis.^4^ Because the study population has been subjected to training in an inadequately organised programme under inexperienced staff, this may have caused them not to want to participate in assessing a programme that had not met their expectations.

However this study provides an important evaluation of the Botswana FM residency programme from the residents’ perspective. Therefore, it contributes to the ongoing discussions about how the Botswana FM residency programme can be improved. Future evaluations of the programme may wish to address the extent to which the findings of this study influenced the development of the Botswana FM residency programme and the health of the population of Botswana. The programme seems to have educational management problem, faculty development issues and scarce resources.

## Lessons learned

Following are lessons to learn from this programme evaluation:

- There is a need for the MOH to define a clear career path for graduates to avoid residents’ despair. This has since been addressed to a significant extent by the MOH.
- Staff employment and development is crucial in new training programme of FM and this is being done.
- Solutions or suggestions to improvement can emanate from residents in a training programme.
- There is a need for involving all stakeholders in implementing suggested solutions, while the Department of Family Medicine should consider empowering willing graduates to join the department in teaching, as recruiting from outside has proved to be difficult.

We recommend faculty development and regional collaboration, which may help the current faculty members to mature and gain required experience for quality teaching and supervision.

## Conclusion

Respondent residents were generally not satisfied with the FM training programme possibly because of the shortage of staff, programme organisational issues and many other related to these. The only areas of satisfaction with the programme appeared to be the weekly tutorial, diversity of patients and diagnosis in Maun and Mahalapye teaching sites. Residents also suggested an increase in faculty staff, the acquisition of equipment for teaching sites and emphasis on FM core topics in the curriculum.

This study contributes to the body of knowledge of FM by providing potential solutions to possible challenges that any new regional FM training programme in a similar situation would have. This contributes to the discussion on the promotion of FM training in the region and in Africa.

Further evaluations of the FM training as well as the assessment of the influence of this training on the health of the Botswana population are needed and such evaluations may improve the FM programme at the UB.

Regardless of the general dissatisfaction with the FM programme, there is hope in addressing the challenges if provided solutions are considered for use by different stakeholders.
